# Protein Aggregation in the ER: Calm behind the Storm

**DOI:** 10.3390/cells10123337

**Published:** 2021-11-28

**Authors:** Haisen Li, Shengyi Sun

**Affiliations:** 1 Center for Molecular Medicine and Genetics, Wayne State University School of Medicine, Detroit, MI 48201, USA; lihs126@163.com; 2 Department of Biochemistry, Microbiology and Immunology, Wayne State University School of Medicine, Detroit, MI 48201, USA

**Keywords:** ER, unfolded protein response, ER-associated protein degradation, ER-phagy, chaperone, protein aggregate, ER storage disease

## Abstract

As one of the largest organelles in eukaryotic cells, the endoplasmic reticulum (ER) plays a vital role in the synthesis, folding, and assembly of secretory and membrane proteins. To maintain its homeostasis, the ER is equipped with an elaborate network of protein folding chaperones and multiple quality control pathways whose cooperative actions safeguard the fidelity of protein biogenesis. However, due to genetic abnormalities, the error-prone nature of protein folding and assembly, and/or defects or limited capacities of the protein quality control systems, nascent proteins may become misfolded and fail to exit the ER. If not cleared efficiently, the progressive accumulation of misfolded proteins within the ER may result in the formation of toxic protein aggregates, leading to the so-called “ER storage diseases”. In this review, we first summarize our current understanding of the protein folding and quality control networks in the ER, including chaperones, unfolded protein response (UPR), ER-associated protein degradation (ERAD), and ER-selective autophagy (ER-phagy). We then survey recent research progress on a few ER storage diseases, with a focus on the role of ER quality control in the disease etiology, followed by a discussion on outstanding questions and emerging concepts in the field.

## 1. Introduction

The ER plays a number of important cellular functions, including the synthesis, processing, and trafficking of proteins and lipids, calcium storage, organelle interactions, carbohydrate metabolism, and detoxification [1,2]. The ER consists of membrane-bound branching tubules and flat sheets, and is categorized into two major types, namely rough and smooth ER, based on their morphological and functional differences [3]. The rough ER is characterized by the presence of ribosomes on the cytosolic side, and functions as the main site for the synthesis and processing of membrane and secretory proteins. The smooth ER is defined by the absence of ribosomes, and functions in the metabolism of lipid and carbohydrate, as well as calcium storage [4]. The shape and composition of the ER are dynamic and undergo rearrangement in response to cellular cues and stresses [1,5,6]. In addition, the ratio of rough to smooth ER is highly diverse in different cell types, reflecting their functional differences. For example, muscle cells have a high content of smooth ER for calcium storage, while B cells and pancreatic acinar cells contain predominantly rough ER for generating secretory proteins [6]. Moreover, the ER keeps in close contact with many other organelles, such as mitochondria, lysosomes, lipid droplets, and Golgi via membrane contact sites, which in turn modulate lipid metabolism, calcium signaling, energy production, protein degradation, and other cellular activities [7,8,9]. Hence, the ER is a central player in the regulation of physiological processes of eukaryotic cells.

**Figure 1:**
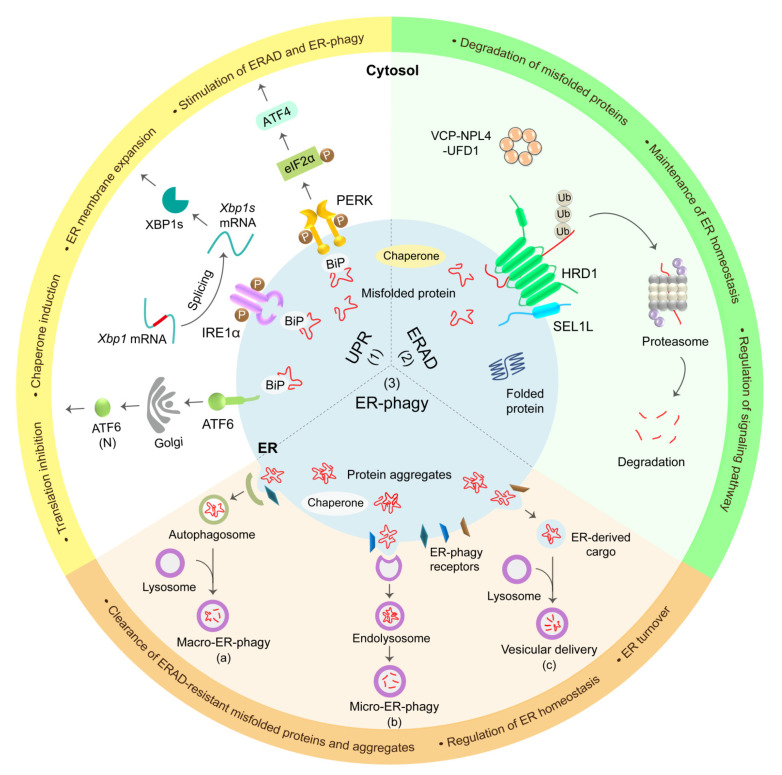
Overview of ER protein quality control system in mammals. (**1**) UPR: misfolded proteins activate three UPR sensors, protein kinase RNA (PKR)-like ER kinase (PERK), inositol-requiring protein 1α (IRE1α), and activating transcription factor 6 (ATF6), which subsequently initiate their downstream signaling pathways to inhibit protein translation, increase ER folding and degradation capacity, promote ER biogenesis, and if the stress is unresolved, trigger cell death. (**2**) ERAD: misfolded proteins are recruited to the ERAD complex for degradation. ER chaperones and lectins mediate the substrate recognition and recruitment to the SEL1L-HRD1 ERAD protein complex. Assisted by AAA-ATPase valosin-containing protein (VCP) and other shuttling factors, ubiquitinated ERAD substrates are delivered to the proteasome for degradation. (**3**) ER-phagy: insoluble protein aggregates in the ER can be cleared via three ER-phagy pathways: (**a**) macro-ER-phagy mediated by autophagosome formation and its subsequent fusion with lysosome for degradation; (**b**) micro-ER-phagy; or (**c**) LC3-dependent vesicular delivery mediated by the engulfment of ER portion by endolysosomes or direct fusion of ER vesicles with lysosomes, respectively. Membrane-bound or soluble ER-phagy receptors define the specificity of ER-phagy. Under pathological conditions, an insufficient activity of ERAD and ER-phagy may lead to elevated protein misfolding and aggregation, thus causing the activation of UPR.

Around one-third of the eukaryotic proteome, including secretory and nearly all membrane proteins, are synthesized in the ER [10,11]. Following their entry into the ER, nascent proteins launch a sequential folding process, along with post-translational modifications such as N-linked glycosylation, disulfide bond formation, and oligomerization to achieve their native and functional conformation [12,13,14]. These events are assisted by various ER chaperones and folding enzymes. However, a significant portion of nascent proteins cannot achieve their native conformation, which are eventually eliminated by proteasomal or lysosomal degradation [12,15]. For instance, it is estimated that 10–15% of newly synthesized proinsulin proteins are misfolded and degraded under normal physiological conditions [16]. To maintain the proteostasis and prevent the accumulation of misfolded proteins in the ER, three protein quality control systems including UPR, ERAD, and ER-phagy are employed to monitor protein biogenesis and dispose faulty proteins in the ER (Figure 1) [15,17]. These protein quality control pathways all work in concert to maintain ER protein homeostasis.

Under pathological circumstances, dysregulated protein folding, transport, degradation, or imbalanced protein synthesis and secretory demand may disturb ER homeostasis, thereby contributing to the disease pathogenesis ranging from metabolic diseases, inflammation, cancer to neurodegeneration [18,19]. In addition, ER stress may be induced in response to the presence of cytoplasmic protein aggregates, thus contributing to neurodegenerative diseases such as Parkinson disease, Alzheimer disease, and Huntington disease [20,21,22]. Moreover, misfolded secretory proteins or aggregates may directly accumulate in the ER, and in some cases, may lead to the development of ER storage diseases [23,24,25,26]. ER storage diseases are often genetically based, and represent heterogeneous clinical phenotypes resulting from the deficiency of the mutant protein in question and/or the toxic effects of misfolded proteins or aggregates in specific cell types [23,24,25,26]. In this review, we will first present a brief overview of the mammalian ER protein folding and quality control systems, followed by discussions of several clinically relevant ER storage diseases, with a focus on the roles of ER protein quality control in disease pathogenesis.

## 2. ER Protein Folding and Quality Control Systems

### 2.1. ER Chaperones

In mammalian cells, ER-targeting proteins contain the ER signal sequence at their amino-terminus, and are co-translationally delivered into the ER via the Sec61 translocon complex on the ER membrane [27,28]. Soluble ER-directed proteins are imported into the ER lumen, while membrane proteins are integrated into the ER lipid bilayer [14]. Newly synthesized proteins moving into the ER undergo sequential modifications and folding to achieve their native conformation with the help of ER chaperones and folding enzymes. Given that ER chaperones have been comprehensively reviewed recently [14,29,30,31,32,33], here we only briefly introduce a few major ER chaperones and folding enzymes.

Binding immunoglobulin protein (BiP), a heat stock protein (HSP) 70 chaperone family member, is one of the most abundant ER chaperones and plays a critical role in protein folding in the ER [31]. BiP possesses a substrate-binding domain that interacts with the exposed hydrophobic segments of client proteins, and a nucleotide-binding domain that binds and hydrolyzes ATP to control the substrate-binding affinity [14,34,35]. Upon the hydrolysis of ATP, ADP-bound BiP exhibits a high affinity for client proteins, thereby allowing the efficient folding of client proteins and shielding them from aggregation. The activity of BiP is further regulated by members of the HSP40 family of ER-localized DnaJ cochaperones (ERdjs) and HSP110 family of nucleotide exchange factors (NEFs). ERdj proteins recruit unfolded client proteins to the ATP-bound BiP and simultaneously trigger ATP hydrolysis [36,37]. NEFs, such as glucose-regulated protein (GRP) 170 and BiP-associated protein (BAP), stimulate the exchange of ADP to ATP, therefore liberating the substrate from BiP [38,39]. Additional post-translational modifications, such as AMPylation, oligomerization and oxidation, have also been reported to regulate the activity of BiP in response to stress and unfolded protein load [40,41,42,43,44]. In addition to assisting protein folding, BiP plays a role in the activation and function of other ER quality control mechanisms such as UPR, ERAD, and ER-phagy as discussed below.

Glycosylation is a key protein modification essential for most secreted and membrane proteins, and plays critical roles in the regulation of protein folding, quality control and activity [14]. The N-linked glycan is pre-assembled of three glucose, nine mannose and two N-acetyl glucosamine residues (Glc_3_Man_9_GlcNAc_2_), and mostly co-translationally transferred to the asparagine residues of newly synthesized polypeptide chains by the oligosaccharyltransferase (OST) [45]. Soon after addition, the N-glycan is rapidly trimmed by ER luminal glucosidases I and II to form monoglucosylated glycan (Glc_1_Man_9_GlcNAc_2_) that is recognized by ER lectin chaperones calreticulin and calnexin [46,47]. Calreticulin is an ER-resident soluble protein, while calnexin is a transmembrane protein with a glycan binding domain in the ER lumen. Their activities as protein folding chaperones are modulated by the glycan composition of maturing glycoproteins in the ER. Once the last glucose is trimmed from the N-glycan, calreticulin and calnexin are released, which allows natively folded glycoproteins to exit from the ER [48,49]. However, if a glycoprotein is not properly folded after a single round of the calreticulin/calnexin cycle, the folding sensor UDP-glucose:glycoprotein glucosyltransferase (UGGT) adds back a single glucose to the N-glycan. The regenerated monoglucosylated protein can be subjected to additional calreticulin/calnexin-mediated folding cycles [50,51,52]. In addition to calreticulin and calnexin, other ER lectin chaperones include osteosarcoma amplified 9 (OS9), XTP3-transactivated gene B protein (XTP3-B), and ER degradation-enhancing mannosidases (EDEM), all of which participate in clearing terminally misfolded glycoproteins via ERAD, which will be discussed later.

The formation of disulfide bonds between cysteine residues is another crucial step for most ER proteins to gain their proper conformation and function; and the formation of aberrant disulfide bonds may result in misfolded proteins and aggregates [53,54,55]. Disulfide bond formation is catalyzed by the oxidoreductase activity of protein disulfide isomerases (PDI). More than twenty oxidoreductases of the PDI family have been identified in mammalian ER to mediate the formation, isomerization, or reduction of disulfide bonds [14,56]. These PDIs display various redox capacities, substrate specificities, chaperone activities, and other important functions, such as UPR signaling and ERAD [56,57]. PDIA1 (also known as PDI) and ERp57 are two most studied PDIs. PDIA1 is the most abundant oxidoreductase accounting for almost 2% of all ER proteins [58], and uniquely exhibits both oxidoreductase and chaperone activities [59]. ERp57 specifically promotes the folding of glycoproteins via the association with both calreticulin and calnexin [60,61]. Another ER-resident disulfide reductase, ERdj5, works in concert with BiP and EDEM1 to reduce non-native disulfide bonds in misfolded proteins and protein aggregates to facilitate their degradation via ERAD [62,63]. As essential players in the folding and quality control of ER proteins, the function, specificity, and mechanisms of many PDIs are still being investigated.
**Keypoints: ER Chaperones**ER chaperones such as BiP, lectin chaperones and PDI cooperate to control the folding and glycosylation of nascent proteins.The function, specificity and mechanisms of many ER chaperones remain unclear.

### 2.2. UPR

When proteins fail to fold properly in the ER, an orchestrated signaling cascade network known as UPR is activated. The overall outcome of UPR is to mitigate global protein translation, promote ER biogenesis, enhance ER folding and degradation capacities, and if the stress is unresolved, trigger cell death [64]. UPR is initiated by three principal ER-resident stress sensors in mammals, inositol-requiring protein 1α (IRE1α), protein kinase RNA (PKR)-like ER kinase (PERK) and activating transcription factor 6 (ATF6) [64]. During basal conditions, BiP binds to the luminal domains of these UPR sensors and keeps them inactive. Under ER stress, BiP dissociates from these sensors, leading to their activation [65,66]. In addition, misfolded proteins may be able to directly interact with and stimulate the UPR sensors such as IRE1α and PERK [67,68,69].

The most conserved UPR sensor IRE1α is a single-span transmembrane protein with a molecular weight of ~110 kDa, and possesses both kinase and endoribonuclease activities within its cytoplasmic domain [70]. Upon activation, IRE1α undergoes dimer- or oligomerization and trans-autophosphorylation, which subsequently stimulates its endoribonuclease activity. IRE1α then excises 26 nucleotides from X-box binding protein 1 (*Xbp1*) mRNA, leading to the generation of an active transcription factor known as spliced XBP1 (XBP1s) [71,72,73]. XBP1s potently induces the expression of genes related to ER protein folding and degradation, as well as lipid metabolism for ER membrane expansion [74]. In addition to *Xbp1* splicing, the endoribonuclease activity of IRE1α may also elicit a process called regulated IRE1α-dependent decay (RIDD) by cleaving other mRNAs and precursor miRNAs as a mean to reduce protein translation and folding load in the ER [75]. However, the physiopathological significance of RIDD remains to be established. It has been proposed that, under prolonged ER stress, IRE1α may promote apoptosis via its RIDD activity, or by activating c-Jun N-terminal kinase (JNK) and nuclear factor-κB (NF-κB) signaling pathways [76,77,78].

Similar to IRE1α, PERK is also a single-span transmembrane protein with a molecular weight of ~150 kDa, comprised of a large ER luminal stress-sensing domain and a cytosolic kinase domain [79]. Once activated, PERK undergoes dimerization and trans-autophosphorylation to activate its kinase domain. The activated PERK subsequently phosphorylates the cytosolic eukaryotic translation initiation factor 2 (eIF2α), leading to attenuated global protein translation and simultaneous translation of activating transcription factor 4 (ATF4) [79,80]. ATF4 activates the transcription of genes involved in protein folding, amino-acid metabolism, autophagy and antioxidant responses [81,82]. Notably, ATF4 induces the expression of pro-apoptotic factor CCAAT/enhancer-binding protein (C/EBP) homologous protein (CHOP) [82,83,84].

ATF6 is also a single-span transmembrane protein with a molecular weight of ~90 kDa, and upon activation, translocates from the ER to the Golgi apparatus where it undergoes proteolytic cleavage by site-1 and -2 proteases to generate a 50 kDa cytosolic fragment of ATF6, called ATF6(N) [85,86]. ATF6(N) enters the nucleus to regulate the expressions of genes encoding ER chaperones, calcium transporters, and ERAD [87]. Interestingly, ATF6 may directly associate with XBP1s to regulate its activity and modulate gene transcription [88,89,90].
**Keypoints: UPR**UPR comprises three distinct signaling cascades initiated by IRE1α, PERK and ATF6 respectively, whose activation inhibits translation, boosts ER folding and degradation capacity, promotes ER biogenesis, and if the stress is unresolved, triggers cell death.The significance and molecular mechanism of the UPR in normal physiology and disease pathogenesis need to be further explored.

### 2.3. ERAD

Misfolded ER proteins are mostly degraded by the cytoplasmic proteasomes due to the lack of protein degradation machinery within the ER. This process is enabled by the process known as ERAD, a highly conserved mechanism that mediates the recognition, retrotranslocation, ubiquitination, and degradation of terminally misfolded ER proteins [12].

Many aforementioned ER chaperones, including BiP, ERdjs, and PDIs, are involved in the recognition and recruitment of misfolded proteins for ERAD. They recognize the conformation and modulate the post-translational modifications of nascent client proteins, thus directing their maturation process. BiP, with the assistance from ERdjs, facilitates the recruitment of non-glycosylated proteins with aberrant conformation or exposed hydrophobic patches to the ERAD machinery [63,91,92,93]. For the glycosylated proteins, the terminal mannose of the N-glycan can be sequentially trimmed by ER mannosidases I and EDEM [94,95,96,97,98]. Lectins, such as OS9 and XTP3-B, then recognize the resulting trimmed glycan on the misfolded glycoproteins and deliver them to the ERAD machinery [99,100,101]. For those proteins with disulfide bonds, PDIs may aid in the recruitment and retrotranslocation processes by reducing the disulfide bonds [102,103]. It remains unclear how these processes are coordinated for nascent misfolded protein substrates.

Following the recognition and recruitment, misfolded proteins are retrotranslocated across the ER membrane via translocon channels, where they can be ubiquitinated by the ERAD E3 ligases. During the retrotranslocation, valosin-containing protein (VCP, also known as p97), a cytosolic AAA-ATPase, provides energy to extract ERAD substrates, while additional shuttling factors such as ubiquitin fusion degradation protein 1 (UFD1) and nuclear protein localization protein 4 (NPL4) facilitate the delivery of ubiquitinated substrates to the proteasome for degradation [104,105,106]. In mammals, over a dozen ERAD complexes with E3 ligase activity have been identified, yet for most of them, the physiological importance and substrate specificity remain unexplored [107,108,109,110,111]. Here we will focus on the best-characterized ERAD complex in mammals, composed of the E3 ligase hydroxymethylglutaryl reductase degradation protein 1 (HRD1) and its cofactor suppressor/enhancer of lin-12-like (SEL1L) [112]. HRD1 functions as not only an E3 ligase, but also a translocon channel with potential assistance from degradation in endoplasmic reticulum protein (Derlin) family members [113,114,115].

As expected, many disease mutants, such as α1-antitrypsin, transthyretin, cystic fibrosis transmembrane conductance regulator (CFTR), and proinsulin are misfolded and degraded by mammalian ERAD [116,117,118,119]. Recent studies have shown that many endogenous proteins such as CD147, lipoprotein lipase (LPL), prohormones proopiomelanocortin (POMC) and vasopressin (pro-AVP) are misfolding prone and bona fide SEL1L-HRD1 ERAD substrates [53,54,120,121]. In the absence of ERAD, these proteins are trapped in the ER, some forming disulfide bond-mediated high molecular weight complexes (LPL, POMC and proAVP) and exhibiting a loss-of-function phenotype [53,54,120]. Moreover, SEL1L-HRD1 ERAD controls the abundance of proteins such as pre-B cell receptor protein (pre-BCR), cAMP responsive element-binding protein, hepatocyte specific (CREBH), IRE1α, nuclear factor erythroid 2-related factor 2 (NRF2), and B lymphocyte-induced maturation protein-1 (Blimp1) [122,123,124,125,126,127,128]. Unlike the first group of substrates, these proteins accumulate in the absence of ERAD, exhibiting a gain-of-function phenotype likely as a result of enhanced re-folding effort. Hence, the fate of the substrates in the absence of ERAD is substrate specific, likely controlled by the chemical and structural property of the substrate. In the next several years, more endogenous substrates will emerge, which will improve our understanding of the significance of ERAD in various physiological processes and human diseases.
**Keypoints: ERAD**SEL1L-HRD1 ERAD is the best-characterized branch disposing both misfolded and native ER proteins via cytosolic proteasomes.The significances of ERAD in various physiological processes and human diseases remain vague.

### 2.4. ER-Phagy

Not all misfolded ER proteins can be degraded by ERAD, due to the limitation of the size of the retrotranslocation pore. ER-phagy, the autophagy/lysosome-mediated selective degradation of the ER domains or fragments, is being increasingly recognized as an alternative ER disposal pathway for ERAD-resistant misfolded proteins or aggregates [2]. The ER-phagy process is enabled by ER-phagy receptors that selectively link ER subdomains to ATG8 proteins LC3 or GABARAP [129,130]. In mammals, six ER membrane-integrated (RTN3, FAM134B, CCPG1, SEC62, TEX264, and ATL3) and three soluble ER-phagy receptors (C53, CALCOCO1, and p62) have been identified so far, all of which contain one or more ATG8-binding regions [2]. TEX264, SEC62, FAM134B, RTN3, and ATL3 are ubiquitously expressed, whereas CCPG1 is predominantly expressed in the liver, kidney, and pancreases [131,132,133,134]. While different ER-phagy receptors may be present on distinct ER subdomains, they may mediate different types of ER-phagy [2].

Based on how ER portions are delivered to the lysosomes, ER-phagy can be classified into three types, termed macro-ER-phagy, micro-ER-phagy, and LC3-dependent vesicular delivery [2,135]. Macro-ER-phagy is characterized by the recruitment of double-membrane autophagosomes and requires both LC3 lipidation and autophagosome biogenesis machineries [2,135]. A recent study showed that procollagen is misfolding prone and resistant to ERAD-mediated degradation. Instead, procollagen is sequestered by ER chaperone calnexin and ER-phagy receptor FAM134B into the autophagosomes, and delivered to the lysosome for degradation [136]. Similarly, ER-retained mutant prohormone aggregates such as POMC, *Akita* proinsulin and Pro-AVP mutants are degraded by RTN3-mediated macro-ER-phagy [137]. In contrast, micro-ER-phagy and LC3-dependent vesicular delivery do not need the involvement of autophagosomes. Micro-ER-phagy is defined as the direct engulfment of particular ER subdomains by the endolysosomes, while LC3-dependent vesicular delivery is mediated by the fusion of ER vesicles into lysosomes [138,139]. For example, the degradation of the G610C mutant procollagen is mediated by micro-ER-phagy, in which the COPII-coated ER exit sites containing mutant procollagen aggregates are directly engulfed into the lysosomes [140]. In another case, the protein aggregates and polymers generated by Z variant of α1-antitrypsin (Z-AAT) are degraded by the LC3-dependent vesicular delivery pathway via the direct fusion of ER-derived vesicles with lysosomes [141]. Despite of recent advances, the detailed mechanism and pathophysiological significance of lysosome-mediated ER degradation remain vague.
**Keypoints: ER-phagy**ER-phagy clears ERAD-resistant misfolded proteins and aggregates, and comprises three different types (macro-ER-phagy, micro-ER-phagy, and the vesicular delivery pathway).The detailed mechanism and pathophysiological significance of ER-phagy are unclear.

### 2.5. Crosstalk among the Quality Control Pathways

In the cells, it is believed that all three quality control pathways are likely orchestrated and integrated to ensure ER homeostasis and the folding and maturation of thousands of proteins synthesized in the ER. Networks of ER chaperones help the folding of nascent proteins, and if it fails, the misfolded proteins are sent to the ERAD complex for proteasomal degradation. If ERAD fails to remove misfolded proteins, these misfolded proteins may form aggregates, which are then the substrates of ER-phagy. If not removed efficiently, misfolded proteins can activate UPR to initiate the overall cellular response including the induction of genes involved in ERAD and autophagy. For example, the IRE1α/XBP1s signaling pathway is known to transcriptionally upregulate the expressions of the core ERAD components, such as HRD1, SEL1L, and Derlin-1 [142]. Similarly, both IRE1α/XBP1s and PERK/eIF2α signaling pathways can induce the transcriptional expression of ER-phagy receptors such as FAM134B, TEX264, and CCPG1, as well as various autophagy ATG genes [143,144,145,146]. Therefore, UPR may stimulate ERAD and ER-phagy to maintain the balance of ER proteostasis by enhancing the clearance of misfolded polypeptides and aggregates.

Both ERAD and ER-phagy may feedback to modulate the UPR signaling. The SEL1L-HRD1 ERAD is reported to mediate the degradation of UPR sensor IRE1α, thereby restraining its activity under basal physiological condition [124]. Upon ER stress, IRE1α dissociates from the SEL1L-HRD1 complex to facilitate UPR signaling [124]. *In vitro*, ATF6 is also a substrate of the SEL1L-HRD1 ERAD [147], although the physiological significance of this remains unclear. Meanwhile, ER-phagy may mediate the degradation of IRE1α oligomers during ER stress, thus protecting against intestinal inflammation [148]. During the resolution phase of ER stress, ER-phagy may degrade ER portions containing stress-induced ER chaperones and enzymes via ER-phagy receptors SEC62 and CCPG1-dependent processes, thus allowing the restoration of physiological ER size and function [132,139,149]. While the molecular details and physiological relevance of these findings remain largely unclear, these ER degradation events are coordinated with the UPR to ensure adaptation to cellular and environmental cues.

Both ERAD and ER-phagy are evolved to cope with misfolded proteins in the ER, yet they exhibit different preferences towards distinct substrates. ERAD is considered to clear soluble substrates that can be retrotranslocated, while ER-phagy disposes ERAD-resistant or structural-constrained faulty proteins and protein aggregates. A good example of this phenomenon is the Niemann-Pick type C protein-1 (NPC1) mutant protein, which can be degraded by both ERAD and ER-phagy. The soluble monomers of disease-causing NPC1 I1061T mutant are partially degraded by MARCH6-mediated ERAD [150]. However, a large portion of them forms insoluble aggregates in the ER, which are removed by FAM134B-mediated ER-phagy [150]. As most of the studies on ER-phagy have been performed *in vitro*, it remains unclear whether and how ERAD and ER-phagy activities are coordinated under (patho)-physiological conditions.

## 3. ER Storage Diseases

Under normal physiological conditions, the coordinated actions of the ER protein quality control systems are able to maintain, adjust, and sustain ER proteostasis by promoting protein folding, clearing misfolded proteins or aggregates, and expanding the ER capacity or altering translation rate when needed. Under pathological conditions, certain misfolded proteins caused by genetic mutations escape the surveillance of ER protein quality control networks and accumulate in the ER. They may form large protein aggregates with disulfide bond-mediated crosslinking, leading to the deficiency of the mutant protein in question and/or the gain of toxic effect in specific cell types [12]. To date, a number of human ER storage diseases have been reported (Table 1) [151,152,153,154,155,156,157,158,159,160,161,162,163,164,165,166,167,168,169,170]. In this section, we discuss recent research progresses on a few cases with focus on the roles of ER protein quality control pathways in the disease etiology and pathogenesis.

### 3.1. α1-Antitrypsin Deficiency (AATD)

α1-antitrypsin (AAT, encoded by the *SERPINA1* gene) is a serine protease synthesized mainly in the liver and secreted into the blood circulation to protect the lung from proteolytic damages caused by neutrophil elastase [151]. During its maturation in the ER, nascent AAT protein undergoes chaperone-mediated folding and N-glycosylation as discussed earlier, and eventually acquires its native conformation for secretion.

Many genetic variants in the *SERPINA1* gene have been found in the human population, including the most prevalent Z-AAT variant carrying an E342K point mutation [151,176]. Patients with homozygous Z-AAT variant develop AATD, characterized by the systemic deficiency of functional AAT and pulmonary emphysema [177]. In addition, a portion of these AATD patients develop ER inclusions of Z-AAT in the hepatocytes, which leads to different degrees of liver injury [152]. Interestingly, the hepatocytes derived from AATD patients with severe liver injury exhibit a lower degradation capacity of Z-AAT in comparison to the liver cells from disease-free individuals, supporting the contribution of defective ER degradation pathways in disease pathogenesis [178].

In hepatocytes, approximately 85% of Z-AAT fails to be secreted, with 70% degraded by ERAD and 15% forming ordered polymers and aggregates in the ER [179,180,181]. In fact, misfolded Z-AAT is one of the first substrates found degraded by the ERAD [182,183]. The overexpression of ERAD machineries including ER mannosidases I and HRD1 may promote the degradation of Z-AAT, thereby increasing its solubility and reducing its aggregation and toxicity [117]. Despite the activity of ERAD, Z-AAT has a tendency to polymerize and form insoluble protein aggregates, leading to the formation of inclusion bodies [152]. Z-AAT inclusions are derived from dilated cisterns of ER, yet distinct from the ER as they contain only PDI and KDEL sequence-containing chaperones, but not calnexin [184,185,186]. Interestingly, the overexpression of calnexin has been reported to inhibit Z-AAT inclusion formation and impair ER secretion in cell culture models [184]. These findings suggest that the formation of inclusion bodies may be a cell-protective mechanism to sequester Z-AAT aggregates from the secretory pathway and sustain normal ER functions. To date, the relationship between inclusion bodies and ER, and the pathological role of inclusions in hepatocyte death *in vivo* remains unclear.

In vitro, ER-phagy is able to degrade Z-AAT aggregates [187]. Elevated autophagy activities have been detected in the livers of AATD patients carrying Z-AAT and in transgenic mouse livers expressing Z-AAT [188,189]. The loss of ATG5, a core autophagy factor, attenuates the degradation of Z-AAT and results in the accumulation of intracellular inclusion bodies [190]. FAM134B, in complex with calnexin, is found to be an ER-phagy receptor for Z-AAT and mediates its degradation via the LC3-dependent vesicular delivery [141]. As in most studies autophagy (not specifically ER-phagy) was studied, the role (and the failure) of ER-phagy and the recognition of inclusions in disease pathogenesis remain elusive.

Currently, liver transplantation is the only curative approach to treat AATD patients with severe liver injury [171,191]. Several emerging therapeutic strategies have been recently suggested to target ER protein quality control pathways (Table 2). Boosting the clearance of hepatic Z-AAT aggregates by autophagy inducers or gene overexpression can efficiently alleviate liver damages in pre-clinical animal models of AATD [192,193,194,195]. The administration of autophagy inducer carbamazepine, rapamycin, or norursodeoxycholic acid, is found to decrease intrahepatic Z-AAT accumulation, suppress hepatocellular death, and reduce liver injury in Z-AAT transgenic mice [192,193,194], providing the basis for a phase II clinical trial (NCT01379469). Similarly, hepatocyte-specific overexpression of transcription factor EB (TFEB), a master regulator in autophagy and lysosomal biogenesis, is reported to reduce Z-AAT inclusion bodies and ameliorate liver fibrosis in Z-AAT transgenic mice [195]. Lastly, 4-phenylbutyric acid, a chemical chaperone enhancing protein folding, is shown to facilitate Z-AAT secretion *in* *cellulo*, and increase blood AAT concentration in Z-AAT transgenic mice [196]. It is important to further evaluate the therapeutic effectiveness of these potential approaches in AATD patients in clinical settings. 

### 3.2. Familial Neurohypophyseal Diabetes Insipidus (FNDI)

FNDI is a rare genetic disorder arising from dominant mutations in the *AVP* gene, characterized by polyuria and polydipsia. FNDI is caused by the deficiency of circulating antidiuretic hormone arginine vasopressin (AVP), which is synthesized as a prohormone (pro-AVP) with eight disulfide bonds and one glycosylation site [202]. Pro-AVP protein is synthesized and folded in the ER of hypothalamic AVP neurons, and then transported to the Golgi apparatus, where it is cleaved into a nonapeptide AVP, a carrier protein neurophysin II (NPII), and a C-terminal glycoprotein fragment [202]. The vast majority cases of FNDI are caused by nonsense or missense point mutations in the AVP or NPII coding regions of the *AVP* gene [203,204]. These mutant pro-AVP proteins display faulty conformations, and are retained in the ER as large amyloid-like aggregates. These aggregates are composed of not only mutant pro-AVP, but also wild type pro-AVP proteins, pointing to the dominant negative nature of disease mutations [157,158]. The formation of aberrant disulfide bonds catalyzed by PDI oxidoreductase is likely to mediate pro-AVP aggregation, as the replacement of all 16 cysteines in pro-AVP completely abolishes the self-aggregation of dominant pro-AVP mutants [205].

Both wild type and mutant pro-AVP have been shown to be degraded by ERAD [206]. Recently, it was reported that the SEL1L-HRD1 ERAD mediates the degradation of endogenous pro-AVP protein in the ER [54]. Mice with global or AVP neuron-specific SEL1L deficiency progressively develop the symptoms of diabetes insipidus. In the ERAD-deficient cells, wild type pro-AVP forms high molecular weight aggregates and is retained in the ER. This observation suggests that even wild type pro-AVP protein is misfolding prone, and that ERAD-mediated clearance of misfolded pro-AVP protein is a critical event in the maturation of nascent pro-AVP [54]. In addition, pro-AVP FNDI mutant proteins can also be degraded by SEL1L-HRD1 ERAD; however, when the ERAD system is overwhelmed, these disease-causing mutant proteins accumulate in the ER and interfere with the biogenesis of wild type pro-AVP protein in a dominant-negative manner [54]. Together, this study demonstrates both physiological and pathological importance of SEL1L-HRD1 ERAD in the maturation of nascent pro-AVP proteins.

In FNDI, pro-AVP-containing vesicles have been found to colocalize with autolysosome markers in cell culture experiments and transgenic animal models [172,197,198]. Autophagophores engulfing the pro-AVP aggregates have been observed in AVP neurons of FNDI transgenic mice [172,199]. The autophagy inducer rapamycin can reduce the pro-AVP aggregates in FNDI transgenic mouse models, whereas the lysosomal inhibitor chloroquine has the opposite effect [172] (Table 2). Consistently, another autophagy inducer carbamazepine has been shown to decrease urinary volume up to 90% and raise urinary osmolality clinically in patients with FNDI [207,208,209] (Table 2). *In vitro*, ER-phagy receptor RTN3 has been shown to mediate the degradation of mutant pro-AVP, further supporting the role of ER-phagy in clearing pro-AVP aggregates [137]. While the activity of autophagy is largely considered cytoprotective, a prolonged activation of autophagy may contribute to neuronal cell death [210,211,212]. Many questions remain in this area, most importantly how the ER quality control pathways function together in response to misfolded pro-AVP and why they fail to prevent the accumulation of pro-AVP aggregates in FNDI.

### 3.3. Mutant INS-Gene-Induced Diabetes of Youth (MIDY)

MIDY is a form of early onset diabetes characterized by insulin deficiency in pancreatic β cells, caused by autosomal dominant mutations in the insulin (*INS)* gene [161,162]. Proinsulin contains three domains: N-terminal B and C-terminal A chains, connected with a polypeptide known as the C-peptide [213]. In the ER, three highly conserved interchain and intrachain disulfide bonds are formed. More than 30 mutations in human *INS* gene have been found in MIDY patients, many of which alter cysteine residues thus disrupting normal disulfide pairing, while the rest affect other highly conserved residues [161]. The proinsulin mutations generally affect the folding of proinsulin in the ER, leading to the ER retention and aggregation with wild type proinsulin via aberrant intermolecular disulfide bonds. Consequently, the folding and secretion of wild type proinsulin are impaired, leading to insulin deficiency [214,215,216]. *Akita* proinsulin mutant is the best-characterized variant bearing a substitution of the cysteine at position 7 of the A chain with tyrosine, hence leaving an unpaired cysteine at the B chain [217,218]. Mouse and pig *Akita* models have been generated to mimic MIDY in humans, both of which develop insulin-deficient diabetes [218,219].

Early studies have shown that β cells of *Akita* mice exhibited increased ER stress levels, as evidenced by ER dilation, elevated *Xbp1* splicing, and induction of BiP and CHOP expression [215,216,218,220,221,222]. In *Akita* mice, loss of ATF6 accelerates hyperglycemia and β cell loss, indicating that ATF6 pathway is protective in response to Akita proinsulin [223]. Similarly, the expression of ER chaperones such as P58^IPK^ and PDIA6 has been described as protective to assist the folding of *Akita* mutant [224,225,226]. Moreover, the loss of pro-apoptotic factor CHOP delays the onset of diabetes in *Akita* mice, further confirming the role of UPR in regulating β cell survival and death under a pathological setting [220]. Together, these observations suggest that UPR signaling largely functions to maintain a productive ER folding environment and protect cells from stress caused by *Akita* proinsulin mutant.

Both ERAD and ER-phagy may also be involved in the degradation of *Akita* proinsulin. An increased expression of HRD1 and SEL1L has been observed in pancreatic islets from *Akita* mice, and the SEL1L-HRD1 ERAD indeed mediates the degradation of *Akita* proinsulin mutant [173,221]. Meanwhile, RTN3-mediated ER-phagy may degrade insoluble *Akita* protein aggregates [137]. ER chaperone GRP170 plays a key role in triaging the proinsulin for ERAD-mediated degradation by repressing the formation of detergent-insoluble proinsulin aggregates [137,214]. By doing so, GRP170 may liberate wild type proinsulin from aggregation and promote its secretion. In addition, the augmentation of SEL1L-HRD1 ERAD or autophagy by estrogen or rapamycin, respectively, has been shown to enhance *Akita* proinsulin degradation and alleviate hyperglycemia in *Akita* mice [173,200] (Table 2). Further investigations are warranted to explore additional mechanisms and pathological significance underlying ERAD and ER-phagy and their crosstalk *in vivo*.

### 3.4. Hepatic Fibrinogen Storage Disease (HFSD)

HFSD is characterized by the ER retention and aggregation of mutant and wild type fibrinogens, leading to liver damage, hypofibrinogenemia, and excessive bleeding [174,227,228]. Fibrinogen, secreted by hepatocytes, is essential for hemostasis, blood clotting, wound healing, inflammation, angiogenesis, and many other processes [229,230,231]. Fibrinogen is synthesized as three polypeptide chains, Aα, Bβ, and γ (encoded by *FGA*, *FGB,* and *FGG* genes, respectively), which undergo a complex folding process and form a 340 kDa hexameric complex (AαBβγ)_2_ via 29 disulfide bonds in the ER [232]. It has been reported that the assembly of fibrinogen protein complex in the ER happens in a sequential manner, with heterodimers of Aα-γ or Bβ-γ formed first, followed by the formation of AαBβγ trimer, and lastly hexamer [233]. During the assembly process, fibrinogen chains and complexes are found to interact with several ER chaperones, including calnexin, calreticulin, BiP and ERp57 [233,234]. It is proposed that calnexin and calreticulin may associate with and retain Aα-γ dimer in the ER, in order for Bβ chain to be incorporated for the trimer and hexamer assembly [233]. Meanwhile, ERp57 mediates the final assembly of two trimers into a hexamer [233]. Once properly assembled, fibrinogen leaves the ER, travels into the Golgi apparatus for hydroxylation and sulfation, and eventually is secreted into the blood circulation as the most abundant coagulation factor [163,235].

Over 200 fibrinogen mutations in *FGA*, *FGB,* and *FGG* genes have been identified in patients with congenital fibrinogen disorders [236]. Out of those, eight mutations (one missense mutation in Aα chain, six missense mutations in γ chain, and one in-frame deletion of five amino acids in γ chain) are found to cause HFSD in an autosomal dominant manner [164,174,175,237,238,239,240]. Interestingly, unlike other missense mutations found in congenital fibrinogen disorders, HFSD mutants form intracellular inclusions in the ER [163,241].

Both ERAD and ER-phagy have been implicated in the degradation of fibrinogen. In fibrinogen Aα knockout mice, Bβ and γ chains are synthesized in the hepatocytes, but are neither secreted nor accumulated in hepatocytes, suggesting their intracellular degradation [242]. Indeed, later studies have shown that both ERAD and ER-phagy participate in the degradation of wild type fibrinogen chains *in vitro* [243,244]. A study performed in yeast suggests that ERAD and autophagy may mediate the degradation of the most prevalent HFSD mutant γ-R401W, bearing an arginine to tryptophan mutation in the γ chain [245]. Recently, the application of autophagy-enhancing drug carbamazepine in two HFSD patients has been shown to alleviate liver damage, suggesting that autophagy can be targeted therapeutically in clearing mutant aggregates [201] (Table 2). Although the molecular mechanisms underlying the biogenesis and quality control of the HFSD mutants remain poorly understood, these studies have provided a solid foundation for future therapeutic treatments of HFSD as well as other relevant diseases.

## 4. Conclusions and Perspectives

As a membrane-bound organelle where protein biogenesis takes place, the ER is evolutionarily armed with a complex protein quality control network to ensure proper protein folding. Though studies over the past years have remarkably expanded our understanding in ER protein quality control and ER storage diseases, many key issues remain to be addressed. First, the mechanisms driving protein aggregation and inclusion formation in the ER are poorly understood. Several mechanisms such as overcapacity in synthesis, insufficient or evaded degradation, and defective ER export have been proposed [12,25,112], but the physiological evidence remains lacking. Second, an increasing list of cytosolic and nuclear proteins have recently been found to undergo phase separation and aggregation in response to various stress, signaling, or pathological conditions [246,247,248]. Such phase separation was induced and regulated by intrinsic factors, such as disordered regions, interacting domains, and post-translational modifications, as well as extrinsic factors such as protein concentrations, chaperones, and RNA molecules [247,248]. Whether similar phase separation plays a role in ER protein aggregation remains unclear. Although the ER possesses a distinct environment compared to the cytosol and nucleus, such as a high concentration of calcium and a lack of RNA molecules, many mechanistic insights, and methods from the cytosolic and nuclear protein phase separation may assist future efforts to understand what happens for protein aggregates in the ER.

**Figure 2:**
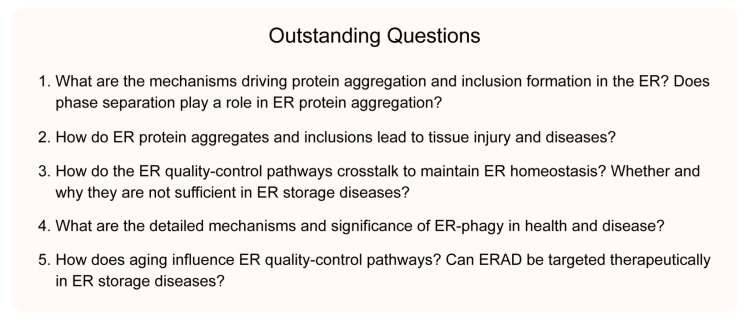
Representative challenging questions related to ER protein quality-control pathways, protein aggregation, and diseases.

Moreover, further investigations are also required to delineate pathological effects of ER protein aggregates and inclusions in various human diseases. In general, the formation of ER protein aggregation has been considered cytotoxic and contributes to tissue damage. For example, the number of hepatic inclusions increases as the liver pathology progresses from fibrosis to cirrhosis in AATD patients, thus a useful hallmark of liver damage [249]. How ER protein aggregates and inclusions lead to tissue injury remains an open question. It was suggested that inclusion bodies might play a protective role by sequestering protein aggregates and sustaining ER function in hepatocytes [184]. Lastly, the molecular details of substrate recognition by ERAD and ER-phagy, and the interplay between ER protein quality control pathways in health and disease, remain vague. Though aging has been described as associated with ER stress [250], the effects of aging on ER folding environment, UPR signaling, ERAD, and ER-phagy activities are poorly understood. These are challenging questions (Figure 2), because they would require the generation and characterization of compound and aged animal models. Nonetheless, probing into these questions will undoubtedly facilitate potential therapeutic strategies to prevent protein aggregates and enhance protein secretion in ER storage diseases.

## Figures and Tables

**Table 1:** ER storage diseases.

Disorder	Protein	Genetic	Pathagenic Variants	Tissue Mutation	Symptoms (Retention)	Ref.
α1-antitrypsin deficiency (AATD)	α1-antitrypsin	Over 100	Recessive	Liver	Hepatic fibrosis Pulmonary emphysema Plasma AAT shortage	[151,171]
Tubulointerstitial kidney disease (ADTKD)	Uromodulin	Over 100	Dominant	Kidney	Gout Hyperuricaemia Low uromodulin	[153,154]
Obesity due to proopiomelanocortin deficiency	Proopiomelanocortin	8 *	Recessive	Hypothalamus	Hyperphagia Insulin resistance Low plasma α-MSH	[155,156]
Congenital hypothyroid goiter with thyroglobulin deficiency	Thyroglobulin	Over 40	Recessive	Thyroid	Deficient thyroid hormone	[159,160]
Mutant INS-gene-induced diabetes of youth (MIDY)	Proinsulin	30 *	Dominant	Pancreas	Hyperglycemia Insulin deficiency	[161,162]
Familial hypercholesterolemia (FH)	Low-density lipoprotein receptor	Over 2000	Dominant	Liver #	Tendon xanthomas Hypercholesterolemia Premature coronary heart disease	[165,166]
Osteogenesis imperfecta (OI)	Procollagen	Over 1500	Dominant	Bone	Osteopenia Multiple fracture Skeletal malformation Short stature	[167,168]
Familial lipoprotein lipase decifiency (LPLD)	Lipoprotein lipase	Over 100	Recessive	Adipose Muscle	Eruptive xanthomas Hypertriglyceridaemia Hepatosplenomegaly Recurrent abdominal pain	[169,170]
Familial neurohypophyseal diabetes insipidus (FNDI)	Vasopressin prohormone	Over 70	Dominant	Hypothalamus	Polyuria Polydipsia Serum AVP deficiency	[172,173]
Hepatic fibrinogen storage disease (HFSD)	Fibrinogen	8	Dominant	Liver	Hemorrhage Hypofibrinogenemia	[174,175]

*: Approximate value; #: Major tissue.

**Table 2:** Potential therapeutic approaches for selective ER storage diseases.

DisorderTreatment		Model	Mechanisms	Effects	Reference
AATD	Carbamazepine	Z-AAT mice	Promotes Z-AAT aggregate degradation Raises autophagy activity	Liver fibrosis ↓	[192]
	Rapamycin	Z-AAT mice	Decreases hepatic Z-AAT accumulation Enhances autophagy activity	Liver fibrosis ↓	[193]
	Norursodeoxycholic acid	Z-AAT mice	Reduces intrahepatic Z-AAT aggregates Boosts autophagic activation	Liver injury ↓ Hepatocellular death ↓	[194]
	TFEB overexpression	Z-AAT mice	Enhances Z-AAT polymer degradation Increases autophagy flux Inhibits Z-AAT expression	Liver fibrosis ↓ Hepatic apoptosis ↓	[195]
	4-phenylbutyric acid	Z-AAT mice	Augments Z-AAT secretion Facilitates protein folding	Blood AAT level ↑	[196]
FNDI	Rapamycin	FNDI mice	Reduces mutant pro-AVP aggregates Increases autophagy flux	Unclear	[172]
	Carbamazepine	Patients with FNDI	Unclear	Urine volume ↓ Urine osmolality ↑	[197,198,199]
MIDY	Estrogen	*Akita* mice	Increases *Akita* proinsulin degradation Stabilizes SEL1L-HRD1 ERAD	Hyperglycemia ↓ Insulin secretion ↑	[173]
	Rapamycin	*Akita* mice	Decreases ER stress Boosts autophagy flux	Hyperglycemia ↓ Insulin production ↑ β-cell apoptosis ↓	[200]
HFSD	Carbamazepine	Patients with HFSD	Enhances autophagy	Liver damages ↓ ALT and AST levels ↓	[201]

ALT: Alanine aminotransferase; AST: Aspartate aminotransferase; ↓: Downregulation; ↑: Upregulation.
